# The basis of clinicopathological heterogeneity in TDP-43 proteinopathy

**DOI:** 10.1007/s00401-019-02077-x

**Published:** 2019-09-26

**Authors:** Ito Kawakami, Tetsuaki Arai, Masato Hasegawa

**Affiliations:** 1 Dementia Research Project, Tokyo Metropolitan Institute of Medical Science, 2-1-6, Kamikitazawa, Setagaya-ku, Tokyo, 156-8506 Japan; 2 Department of Neuropathology, Tokyo Metropolitan Geriatric Hospital and Institute, Tokyo, Japan; 3 Department of Psychiatry, Division of Clinical Medicine, Faculty of Medicine, University of Tsukuba, Tsukuba, Japan

**Keywords:** TDP-43, Progression, FTLD, ALS, Vulnerability, Proteinopathy

## Abstract

Transactive response DNA-binding protein 43 kDa (TDP-43) was identified as a major disease-associated component in the brain of patients with amyotrophic lateral sclerosis (ALS), as well as the largest subset of patients with frontotemporal lobar degeneration with ubiquitinated inclusions (FTLD-U), which characteristically exhibits cytoplasmic inclusions that are positive for ubiquitin but negative for tau and α-synuclein. TDP-43 pathology occurs in distinct brain regions, involves disparate brain networks, and features accumulation of misfolded proteins in various cell types and in different neuroanatomical regions. The clinical phenotypes of ALS and FTLD-TDP (FTLD with abnormal intracellular accumulations of TDP-43) correlate with characteristic distribution patterns of the underlying pathology across specific brain regions with disease progression. Recent studies support the idea that pathological protein spreads from neuron to neuron via axonal transport in a hierarchical manner. However, little is known to date about the basis of the selective cellular and regional vulnerability, although the information would have important implications for the development of targeted and personalized therapies. Here, we aim to summarize recent advances in the neuropathology, genetics and animal models of TDP-43 proteinopathy, and their relationship to clinical phenotypes for the underlying selective neuronal and regional susceptibilities. Finally, we attempt to integrate these findings into the emerging picture of TDP-43 proteinopathy, and to highlight key issues for future therapy and research.

## Introduction

The pathology of proteinopathies appears in distinct brain regions, involves disparate brain networks, and features accumulation of misfolded proteins in various cell types and in different neuroanatomical regions. The clinical phenotypes of these diseases correlate with the characteristic distribution patterns of the underlying pathology across specific brain regions with disease progression. Individuals may exhibit rapid or slow neurodegeneration even within the same syndrome. However, little is known to date about the reasons for this, although an understanding of the basis of the selective cellular and regional vulnerability would be helpful for the development of targeted and personalized therapies. Notably, there is growing evidence that these proteinopathies are modulated by genetic risk factors, differences in the conformation of pathological proteins, and various environmental factors. These factors may play a role in differential cellular susceptibility or resilience, and hence result in phenotypic diversity.

Transactive response DNA-binding protein 43 kDa (TDP-43) was identified as a major disease-associated component in the brain of patients with amyotrophic lateral sclerosis (ALS) and the largest subset of patients with frontotemporal lobar degeneration with ubiquitinated inclusions (FTLD-U) [6, 116]. FTLD-U has characteristic cytoplasmic inclusions, which are positive for ubiquitin but negative for tau and α-synuclein. The terminology FTLD-TDP has been recommended for the neuropathological subtype of FTLD characterized by abnormal intracellular accumulations of TDP-43 [44, 48, 99, 102, 114, 143].

In this review, we summarize recent advances in the neuropathology, genetics and animal models of TDP-43 proteinopathy, and discuss their relationship to clinical phenotypes, their place in the emerging picture of TDP-43 proteinopathy, and their relevance to the development of therapeutic interventions.

## Cellular and molecular basis of TDP-43 aggregation

### Structure, function and localization of TDP-43

**Fig. 1:**
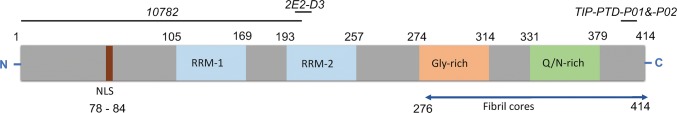
Schematic diagram of TDP-43 functional regions involved in the pathogenesis and progression of TDP proteinopathy. TDP-43 in human brain is a heterogenous ribonucleoprotein with two RNA recognition motifs (RRM-1 and 2) in the middle portion and with a glycine-rich domain and Q/N-rich domain in the C-terminal region. Protein in abnormal conformations is accumulated in brains of patients in the form of amyloid-like fibril structures. The blue arrow indicates the protease-resistant fibril core. Mapping of three selected antibodies to the TDP-43 protein is shown. Antibodies in italics are widely used in TDP-43 immunohistochemical studies [62]. *NLS* nuclear localization signal, *RRM* RNA-recognition motif, *Gly-rich* glycine-rich domain

TDP-43 was originally discovered in a screen for protein factors that bind the long terminal repeat transactive response element of HIV-1 [119]. Several studies have revealed that TDP-43 can bind both DNA and RNA [31, 32, 94]. There is evidence that TDP-43 plays a role in transcription, alternative splicing and messenger RNA (mRNA) stability, and is involved in various cellular processes, including apoptosis, cell division and axonal transport [31, 94]. The capacity of TDP-43 to interact with RNA, including mRNA and pre-mRNA, is considered to underlie the role in exon splicing. TDP-43 is a heterogeneous nuclear ribonucleoprotein with two RNA recognition motifs (RRM-1 and 2) in the middle portion [93] and a glycine-rich domain and glutamine/asparagine (Q/N)-rich domain in the C-terminal region. Most mutations in TDP-43 are clustered in the C-terminal region [87], which is essential for some of the most well-characterized functions of TDP-43, including the protein’s role as a gene splicing factor [119] (Fig. 1).

TDP-43 shuttles between the nucleus and cytoplasm under physiological conditions, and a nuclear localization signal enables the active import of the protein into the nucleus [93, 159]. The majority of TDP-43 resides in the nucleus, but up to 30% of TDP-43 resides in the cytoplasm [17]. Export is mainly driven by passive diffusion, and TDP-43 is predominantly localized in the nucleus in the healthy state [93]. Under pathophysiological conditions, TDP-43 is cleared from the nucleus and accumulates in the cytoplasm [6, 68, 114]. The translocation into the cytoplasm has been considered to be a consequence of impaired nuclear transport and solubility of TDP-43 [159]. This is consistent with findings that seeded aggregation of TDP-43 in cytoplasm may induce the mislocalization of TDP-43 by impairing nuclear transport [17], and that proteins playing a role in nucleocytoplasmic transport are present in pathological TDP-43 aggregates [37].

The neurotoxicity of pathological TDP-43 protein has not been fully clarified. Although the redistribution of TDP-43 from the nucleus to the cytoplasm is generally considered to cause loss of TDP-43 nuclear function [32], some studies have found that nuclear depletion is not necessary for neuronal toxicity induced by ALS-associated mutant TDP-43 [10, 13], and that cytoplasmic mutant TDP-43 has an important role in neurodegeneration [17]. More recent animal model studies support the hypothesis that TDP-43 progression is induced by a gain of toxic function in the cytoplasm, in addition to the loss of nuclear function, and that both are involved in the progression of TDP-43 proteinopathy [50, 51, 57, 138]. Understanding in detail how TDP-43 leads to neurodegeneration will help direct future therapy.

### TDP aggregates and their molecular alterations

**Fig. 2:**
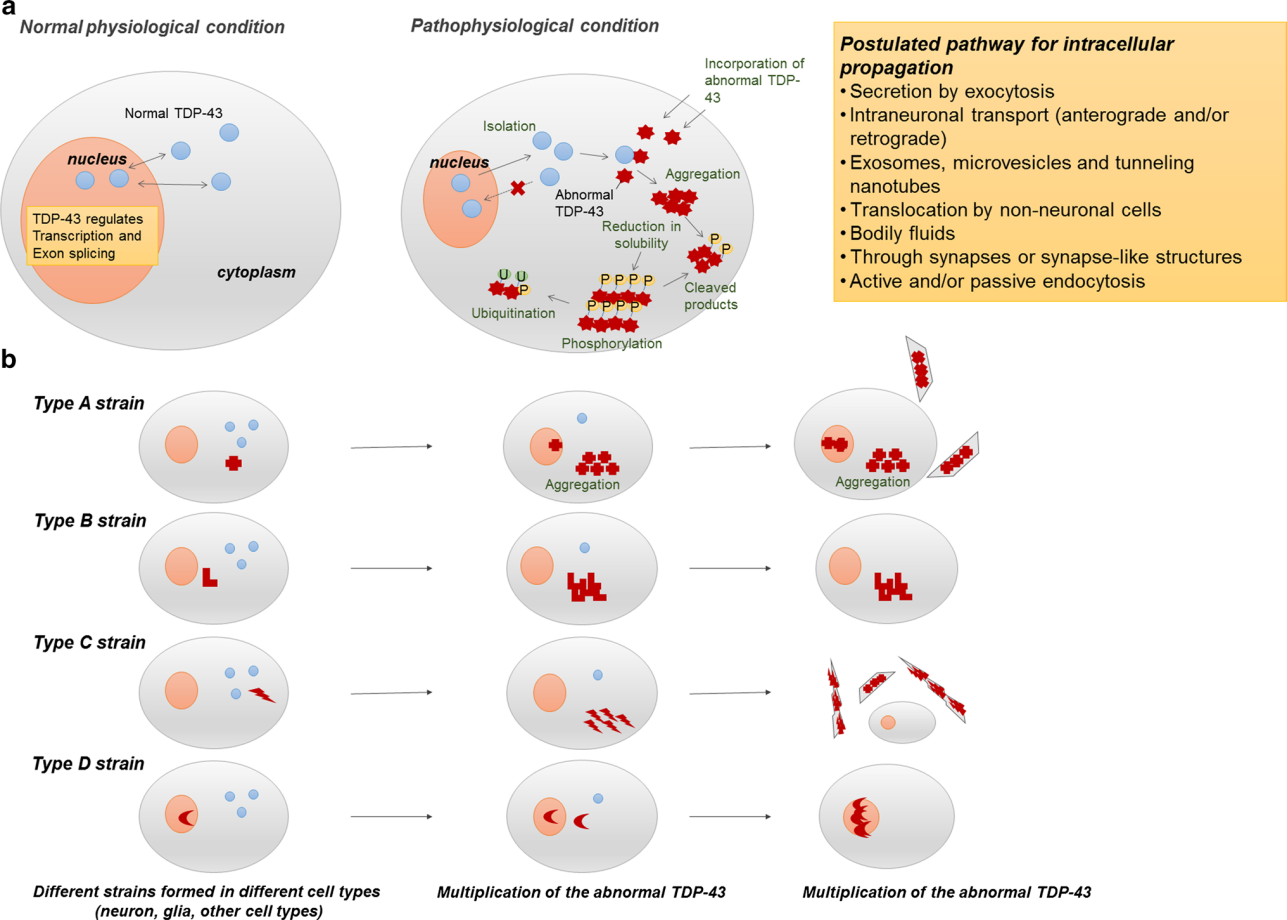
Postulated molecular mechanisms of accumulation and propagation of pTDP-43 in human brain. **a** Normal physiological status and pathophysiological status of TDP-43 in human brain. As shown on the left, TDP-43 primarily resides in the nucleus and is trafficked between the nucleus and cytoplasm in the cells under normal physiological conditions. TDP-43 has roles in regulating gene expression at the transcriptional level, regulating gene splicing, and stabilizing mRNA. Under pathophysiological conditions in patients with pTDP pathology (right), TDP-43 is cleared from the nucleus and accumulates in the cytoplasm. Pathological TDP-43 is fragmented into C-terminal fragments, which are hyperphosphorylated, ubiquitinated and less soluble, and might represent immature products available for aggregation. *P* phosphorylated, *TDP-43* TAR DNA-binding protein 43, *U* ubiquitinated. **b** TDP-43 proteinopathy can involve several strains of seeds. Each pTDP-43 strain accumulates in different cell types and regions of the central nervous system. The strains move from one region to another, maintaining their original form. All of these phenomena may contribute to selective local vulnerability. The expression levels of isoforms of the cognate proteins may differ among cells and compartments, thereby promoting or restricting the spread of the seeds and defining the strain of seed that is propagated

Biochemical, immunochemical and protein-chemical analyses of materials isolated from the brains of patients with TDP-43 proteinopathies have uncovered the characteristic features of pathological TDP-43. Immunoelectron-microscopic studies demonstrated that the protein accumulates as abnormal fibrils or filaments with cellular organelles, irregularly shaped structures and lipofuscins. The fibrils appear to be amyloid-like, even though they are intracellular proteins, whereas the classical histopathological “amyloid” exists as extracellular proteinaceous deposits exhibiting beta sheet structure [6, 68, 116]. More extensive studies demonstrated that the protein is poorly sarkosyl-soluble, hyperphosphorylated, ubiquitinated, and abnormally cleaved into small fragments (C-terminal fragments) [6, 68, 86, 116]. These data indicate that functional consequences might ensue from the distinctive differences between normal and abnormal TDP-43 (Fig. 2a). It has been suggested that aggregation-prone N-terminal fragments produced by proteolytic cleavage may cause TDP-43 pathologies, but TDP-43 cleavage peptides generated by enzymes such as caspase or calpain have not been detected by nano-flow liquid chromatography–ion trap mass spectrometry [86]. Furthermore, aggregation of full-length TDP-43 precedes generation of TDP-43 C-terminal fragments in the seeded cellular model of TDP-43, suggesting that production of C-terminal or N-terminal fragments is not essential for the formation of intracellular TDP-43 aggregates [86].

As in other neurodegenerative diseases, hyperphosphorylation seems to occur in the aggregated TDP-43 proteins. Many phosphorylation sites, including serine, threonine, or tyrosine residues, have been reported within TDP-43 in cellular experiments, and these residues may have some functional role in TDP-43. However, intensive immunochemical studies using more than 20 phosphorylation-site-specific antibodies for the human TDP-43 sequence revealed that several of the antibodies (pS379, pS403/404, pS409, pS410 and pS409/410) stained the pathological TDP-43 inclusions. These antibodies specifically recognize hyperphosphorylated full-length and cleaved forms of abnormal TDP-43, and thus are frequently used for immunostaining and immunoblotting studies [62, 68]. The phosphorylation and fragmentation were recapitulated in a cellular model of seeded aggregation of TDP-43 [117] (Fig. 2a). In addition, recombinant full-length human TDP-43 forms structurally stable, spherical oligomers that are neurotoxic in vitro and in vivo [50]. In fact, cellular aggregate formation or accumulation of TDP-43 C-terminal fragments is not primarily responsible for the development of the observed disease phenotype in mutant or wild-type TDP-43 mice [43]. These findings also suggest that full-length TDP-43, not the cleavage fragments, participates at an early stage in TDP-43 pathology and also fragmentation might occur after the accumulation of TDP-43 [86].

Notably, patients with PGRN gene (GRN) mutations have high frequency of neuronal intranuclear inclusions (NIIs) containing TDP-43. The formation mechanism of NIIs is still unknown, but one possibility is that the production of NIIs might be associated with the deficiency of PGRN, which is involved in the regulation of lysosomal function since this would lead to the increased lysosomal gene expression and protein levels [144, 145] (Fig. 2a).

### Physiological microenvironments associated with neuronal toxicity

Previous research on physiological microenvironments associated with neurodegeneration in TDP-43 proteinopathy has focused on stress granules (SGs). These are membrane-less discrete cytoplasmic structures, containing mRNA and associated proteins that form in cells as a protective response to stress [30]. SGs have long been thought that they contribute to some degenerative diseases, including ALS, FTD, myopathy, and possibly AD and other tauopathies [12, 29, 150]. Many prior studies suggest that TDP-43 and other diseases associated with RNA-binding proteins colocalize with SGs, and SGs might regulate the translational stress response [151]. However, it is still unclear whether TDP pathology arises from SG pathology since colocalization of TDP-43 and SG proteins is hardly observed in human brains [160]. Recent papers demonstrate that cytoplasmic inclusions containing aggregated pTDP-43 can develop through three pathways: direct aggregation or phase-separated intermediates involving ejection from SGs or seeding with exogenous fibrils [58, 105]. These studies suggest that pathological TDP-43 granules are distinct from SGs, although in some cases SG appears to serve as an intermediate. They also indicate that most of the nuclear pore proteins do not colocalize with TDP-43 aggregates, implying that the aggregation occurs through an independent process [58, 105].

### TDP-43 expression in non-neuronal cells

In addition to neurons, TDP-43 is abundantly expressed in glia, as well as many other cell types. Oligodendrocytes with accumulations of pTDP-43 frequently appear in FTLD/ALS brains [5, 26], and TDP-43-positive inclusions in astrocytes have been recognized in Alexander’s disease [152]. Basically, microglia play a role in mediating neurotoxicity and neuroprotection, and maintaining homeostasis, including control of the formation of synapses, refining neuronal circuits, eliminating redundant synapses, and healing responses to injury [139].

Recent studies of mice with TDP-43 pathology induced with TDP-43 from patients with TDP-43 proteinopathy established that white matter oligodendrocytes and astrocytes are capable of developing phosphorylated TDP-43 aggregates [122], and abundant reactive microglia selectively cleared neuronal human TDP-43 [74]. Studies in animal models of other proteinopathies, such as α-synuclein, have also described neurodegeneration with inflammation, and activated microglia [136]. These findings suggest that inclusions or degenerating neurons with aggregates may be cleared by microglial phagocytosis, which may account for the neuroprotective action of microglia [136, 139]. Modulation of microglia-mediated function during the progression of TDP-43 pathology may have potential as a new therapeutic strategy.

## Phenotypic diversity in TDP-43 proteinopathy

### Clinical phenotypic diversity in TDP-43 proteinopathy

Many neurodegenerative diseases, such as Alzheimer’s disease (AD), Parkinson’s disease (PD), and amyotrophic lateral sclerosis (ALS), are classified according to their clinical symptoms and neuropathological features. Indeed, major clinical symptoms correspond well to the anatomical brain regions showing neuronal loss with reactive gliosis. These pathological changes are associated with the accumulation of abnormal proteins with conformational changes and modifications, which occur intracellularly in neurons or glial cells, or extracellularly [89].

ALS and FTLD-TDP are sporadic and familial neurodegenerative diseases characterized neuropathologically by cellular aggregates of TDP-43. The differences in TDP-43 pathology are associated with differences in clinical manifestations, and ALS and FTLD are generally diagnosed on the basis of clinical symptoms. However, some patients may show symptoms common to both diseases. For example, clinical overlap between ALS and behavioral variant frontotemporal dementia (bvFTD) has been recognized, with up to 50% of ALS patients showing some executive function deficits [98, 127]. Also, up to 15% of bvFTD patients show motor neuron dysfunction [34, 127].

**Fig. 3:**
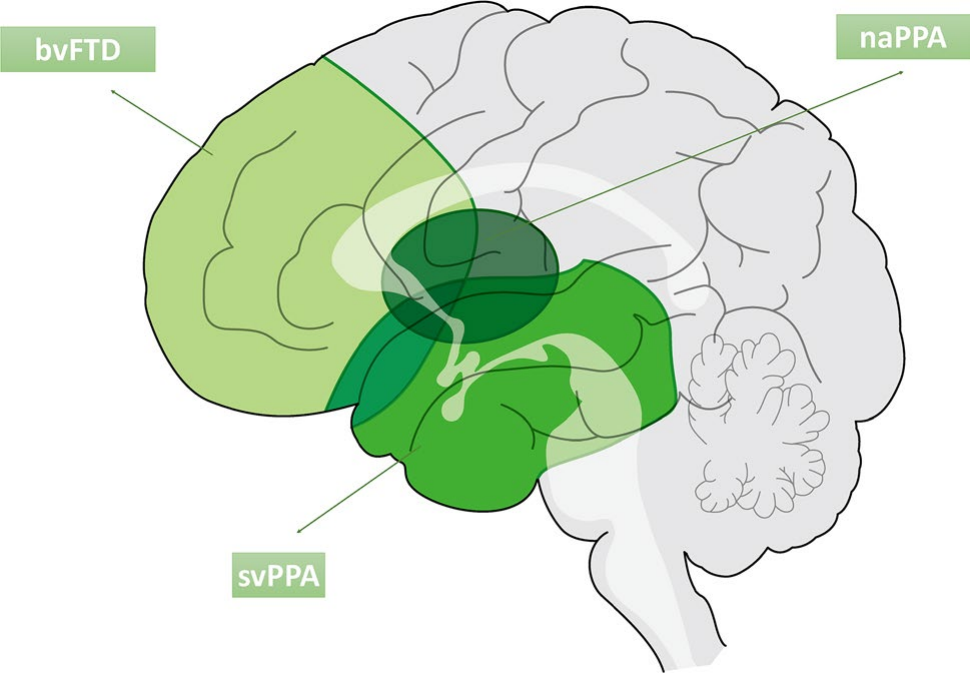
Specific topographic patterns of atrophy in FTLD-TDP. bvFTD predominantly shows frontal lobe atrophy, svPPA shows left anterior temporal tip atrophy, and naPPA shows left frontotemporal atrophy. Each clinical syndrome is associated with distinct cerebral involvement behavioral variant FTD: bvFTD, semantic variant PPA: svPPA, nonfluent/agrammatic PPA: naPPA

There are three major clinical phenotypes of frontotemporal lobar degeneration (FTD): bvFTD, semantic variant primary progressive aphasia (svPPA) and nonfluent/agrammatic primary progressive aphasia (naPPA) [36, 44, 48]. Each clinical syndrome among these diseases is associated with topographically distinct cerebral involvement, based on pathological protein deposits. bvFTD is associated with symmetrical (though sometimes right-sided) frontotemporal dysfunction, svPPA with left anterior temporal deficits and naPPA with left frontotemporal dysfunction (Fig. 3).

Loss of empathy, apathy, and selfishness are more frequent features of bvFTD, but may be found commonly in all subtypes [18]. These symptoms are associated with atrophy and dysfunction of the right anterior cingulate cortex and superior frontal gyrus, while disinhibition is associated with the right subgenual cingulate cortex and orbitofrontal cortex [121, 163], overeating with an orbitofrontal striatal circuit [161], and executive dysfunction with the dorsolateral and prefrontal cortex [73]. Fluent speech, progressive impairment of single-word comprehension, preserved articulatory abilities, and dysfunction of semantic memory are features of svPPA [63]. Patients with naPPA show apraxia of speech or expressive agrammatism: single-word comprehension and object knowledge are relatively preserved, and behavioral symptoms are less common in comparison with their high frequency in svPPA. Cognitive decline, dementia, and alterations in high-order brain functions are associated with the involvement of the limbic region, including the entorhinal cortex and hippocampus, and neocortical areas. Most svPPA can be classified into TDP proteinopathy, whereas bvFTD and naPPA are correlated with FTLD-tau [137].

## TDP-43 pathology in aged brains and other neurodegenerative diseases

In FTLD-TDP and ALS, neuronal loss, reactive gliosis, microglial activation and superficial laminar spongiosis are seen in the neocortex and hippocampus [48]. TDP-43 pathology is present throughout the CNS with relative preservation of the occipital cortex and cerebellum [61]. Subcortical areas, including the nucleus accumbens, basolateral amygdala and caudate/putamen, are also progressively affected [61]. In movement disorders such as ALS, the basal ganglia, thalamus, brainstem nuclei, cerebellar cortex and nuclei, motor cortical areas and lower motor neurons of the spinal cord are involved. These findings suggest that selected neuronal populations may be particularly vulnerable to TDP-43 aggregates. TDP-43 immunoreactive structures include neuronal cytoplasmic inclusions (NCIs), neuronal pre-inclusions, dystrophic neurites (DNs), neuronal intranuclear inclusions (NIIs), and glial cytoplasmic inclusions (GCIs).

The deposited protein is hyperphosphorylated, ubiquitinated and abnormally cleaved to generate C-terminal fragments [6, 68, 116]. The phosphorylation sites of abnormal TDP-43 are located at Ser379, Ser403, Ser404, Ser409 and Ser410. TDP-43 inclusions are thioflavin-S positive [22], suggesting that TDP-43 is accumulated as amyloid-like fibrils.

**Fig. 4:**
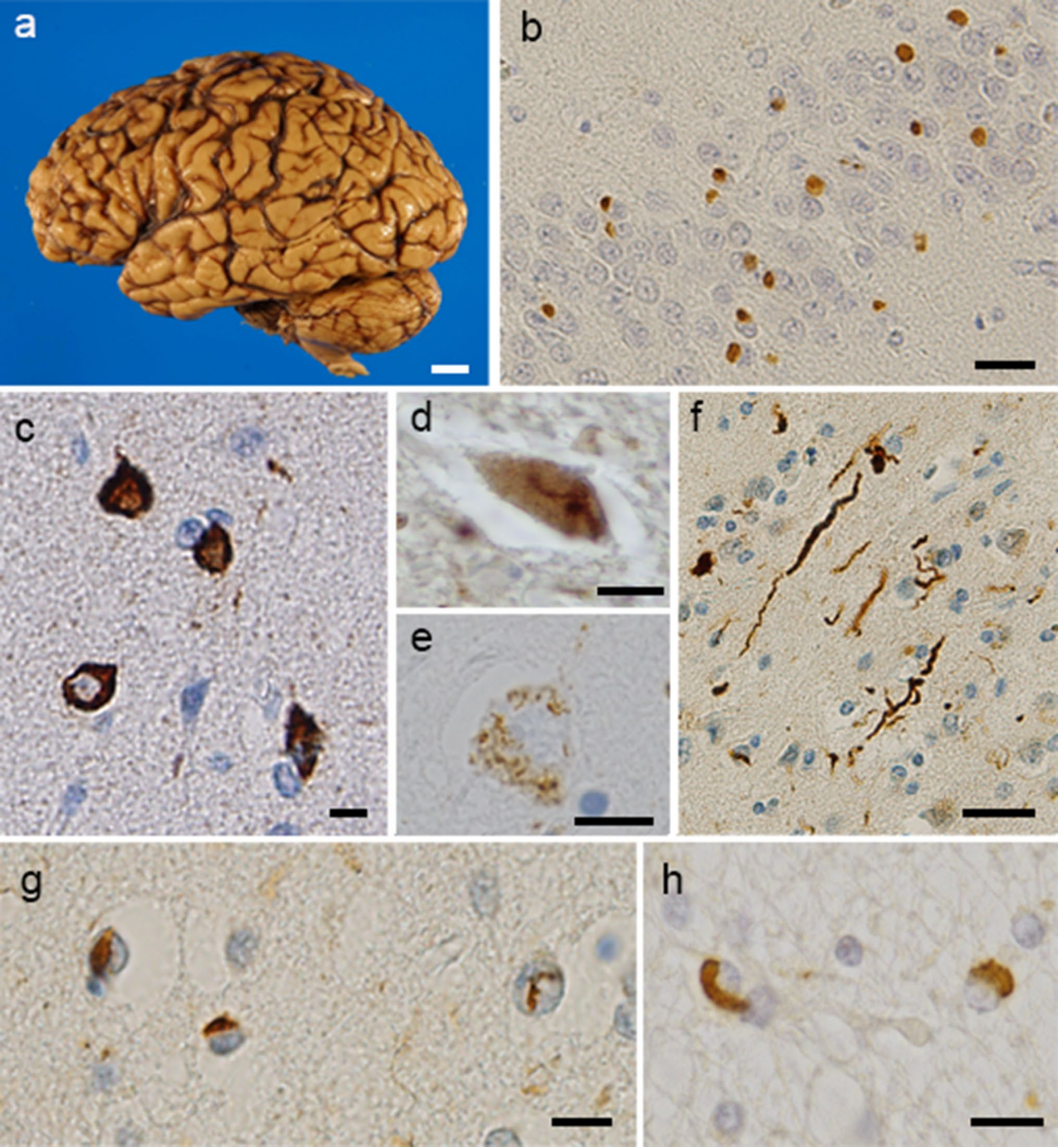
Pathological features of TDP-43-positive structures. Macroscopic photograph of an FTLD-TDP patient’s brain. The left hemisphere shows severe atrophy in the frontal cortex and mild atrophy in the temporal tip (**a**). TDP-43-positive structures detected in patients with TDP proteinopathy. The dentate gyrus of the hippocampus shows neuronal cytoplasmic inclusions (NCI) with no nuclear staining (**b**). Rounded NCI in the entorhinal cortex (**c**), skein-like inclusions in the lower motor neurons (**d**), and inclusions with granular appearance in the amygdala (**e**) can be seen. There are dystrophic neurites (DNs) in the temporal cortex (**f**). Neuronal intranuclear inclusions with a “cat’s eye” shape are present in the entorhinal cortex (**g**) and glial cytoplasmic inclusions are seen in temporal white matter (**h**). **b–h** Stained with phosphorylated TDP-43-specific antibody (pS409/410). Scale bars: **a** 1.5 cm; **b**, **d**, **f** 20 μm; **c**, **e**, **g** 10 μm

TDP-43-positive NCIs are frequently found in the frontotemporal neocortex and dentate granule cells of the hippocampus in ALS, FTLD and FTLD–MND [76, 118, 157]. They are also positive for ubiquitin and p62, but negative for tau, α-synuclein, amyloid beta and FUS [6, 36, 44, 116, 118]. Skein-like and spherical or round inclusions are present in motor neurons in ALS. These inclusions are detected in 100% of TDP proteinopathy brains. Diffuse or granular or dash-like cytoplasmic inclusions, which are dispersed throughout the neuronal somatodendritic domain, are found in motor neurons of cranial nerve nuclei or spinal cord of ALS and FTLD-TDP [44, 60]. These inclusions are considered to be in a transitional state and are referred to as “pre-inclusions”, since they are negative for ubiquitin and p62 [5]. As for DNs, two types of DNs, short DNs and elongated DNs, have been recognized [36, 71]. Both types of DNs are typically most numerous in layer II of the frontal and temporal cortices, although the elongated DNs are generally more widely dispersed throughout the entire cortex in comparison with the short DNs [71]. NIIs show a lentiform or “cat’s eye” appearance and are present in small neurons in multiple neuroanatomical sites. They appear frequently in familial cases [60]. GCIs with coiled body-like or globular morphologies are frequently found in the cerebral white matter and the spinal cord in ALS and FTLD–MND [27, 71, 115]. GCIs are positive for TDP-43 and p62, but are mostly negative for ubiquitin [9, 60]. Most GCIs are considered to be of oligodendrocytic origin on the basis of double immuno-labeling for pTDP-43 and a complement protein, C4d [7] (Fig. 4).

**Fig. 5:**
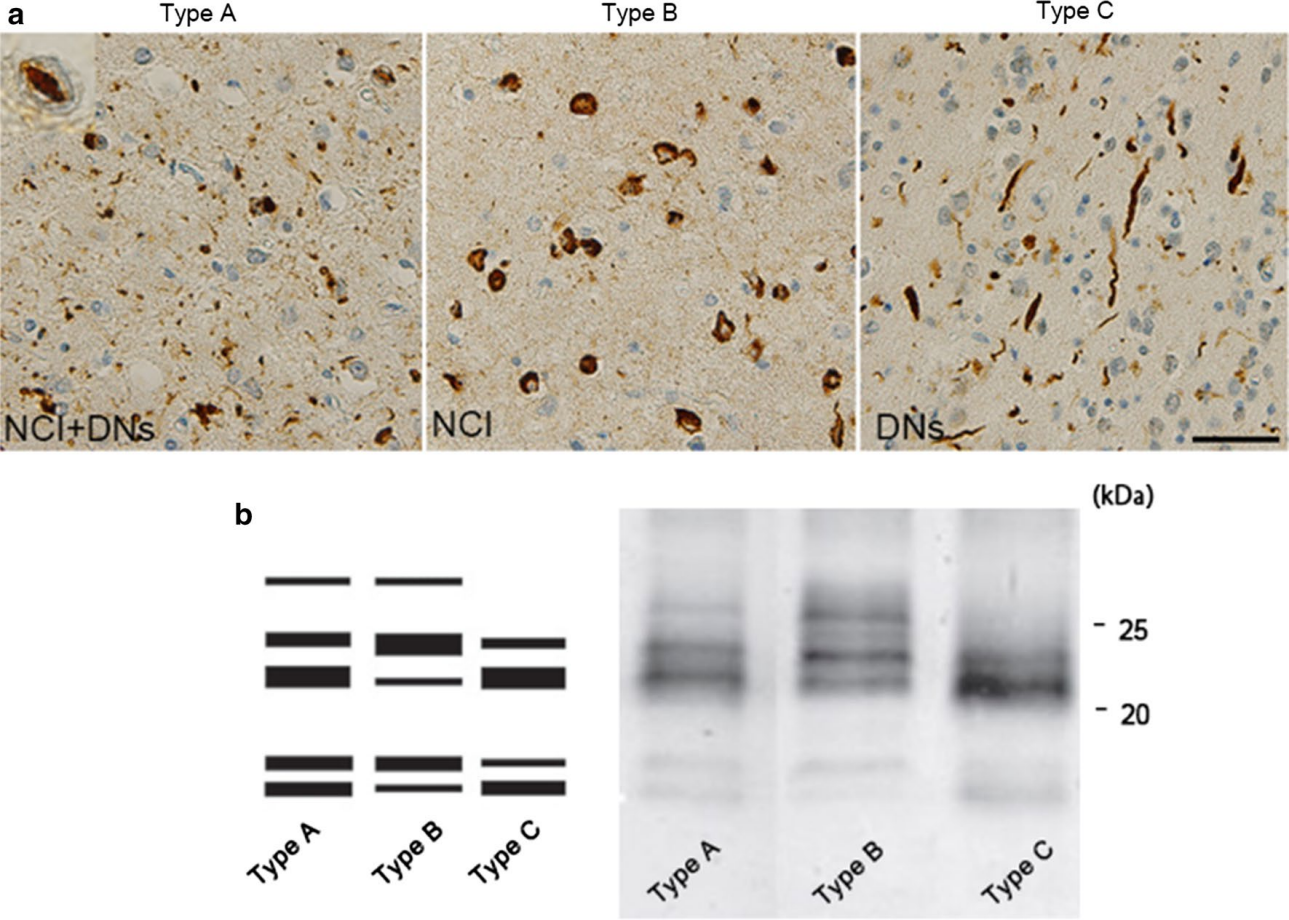
Pathological and biochemical classification of TDP proteinopathy. **a** FTLD-TDP subtypes A to C. Type A is characterized by numerous neuronal cytoplasmic inclusions (NCIs) and short dystrophic neurites (DNs). This type frequently has neuronal intranuclear inclusions (NII) in the affected regions; type B shows numerous NCIs; type C has long and tortuous DNs. **b** Immunoblots of insoluble TDP-43 from patients with TDP proteinopathy, probed using anti-phosphorylated TDP-43 antibody (pS409/410). The band patterns of TDP-43 C-terminal fragments from the three TDP-43 types are different (shown schematically on the left). Type B has three major bands at 23, 24 and 26 kDa and two minor bands at 18 and 19 kDa (lane 2). Type C has two major bands at 23 and 24 kDa and two minor bands at 18 and 19 kDa (lane 3). Type C has an intense band at 23 kDa, whereas type B has a strong 24 kDa band. The band pattern of type A seems intermediate between types B and C (lane 1). Molecular weight markers of migrated protein standards are shown in kDa. Scale bar: 20 μm

TDP-43 pathology is typically classified into four subtypes (types A–D) in FTLD-TDP, based on the predominant type of TDP-43-positive structures and their distribution [101]. Type A is characterized by numerous short DNs and crescentic or oval NCIs; type B shows NCIs throughout all cortical layers with few DNs; type C presents a predominance of elongated DNs in upper cortical layers with few NCIs; and type D is characterized by numerous short DNs and frequent lentiform NIIs (Figs. 2b, 5a). Recently, a new type, type E, was identified, in which granulofilamentous neuronal inclusions, abundant grains, and oligodendroglial inclusions were prominent [95].

TDP pathology should be evaluated from multiple points of view. Neuropathological comorbidity has recently attracted attention. TDP-43 pathology frequently appears in other neurodegenerative diseases, including AD, argyrophilic grain disease (AGD), dementia with Lewy bodies (DLB), chronic traumatic encephalopathy (CTE), hippocampal sclerosis (HS), and Guam ALS [8, 61, 67, 90, 109, 113, 162]. Concomitant TDP-43 pathology has a significant impact on the clinical features of AD [82, 83]. TDP-43 pathology in AD has been classified into six stages, supported by correlations with neuroimaging and clinical features [82, 83]. It begins with the amygdala (stage I), then spreads into the subiculum and entorhinal cortex (stage II), and involves the dentate gyrus and occipitotemporal cortex (stage III). Next, the insular, ventral striatum, basal forebrain and inferior temporal cortex are affected (stage IV). Then it appears in the substantia nigra, inferior olive nucleus, and midbrain tectum (stage V), and finally in the basal ganglia and middle frontal cortex (stage VI) [82, 83]. Progression is associated with impaired cognition, memory loss, and medial temporal atrophy in AD [83]. Concomitant TDP-43 pathology was also found in 53–60% of DLB cases, 60% of AGD cases (Fig. 3e) and 100% of Huntington’s disease cases [8, 57, 130]. TDP pathology was seen in 89.9% of patients with HS compared with 9.7% of patients without HS [113]. TDP-positive inclusions and neurites were also found in 36–85% of CTE cases, ranging from early to late stage [109].

TDP-43 pathology is present in a high proportion of cognitively normal elderly persons [11]. In cases of primary age-related tauopathy (PART), which is characterized by the presence of neurofibrillary tangles (NFTs) in the limbic and brain stem region and absent-minimal β-amyloid deposition [41], 26.7% have TDP-43 pathology and the presence of TDP-43 was associated with significantly greater amygdala, hippocampal, and anterior temporal atrophy [80].

As we described above, TDP-43 pathology frequently occurs in AD and DLB cases. In 20–30% of these cases, the morphological pattern of cortical TDP-43 immunoreactivity and the band pattern of C-terminal fragments of insoluble TDP-43 on immunoblots are similar to those of type A FTLD-TDP, which is seen in cases with *GRN* mutation [8].

**Fig. 6:**
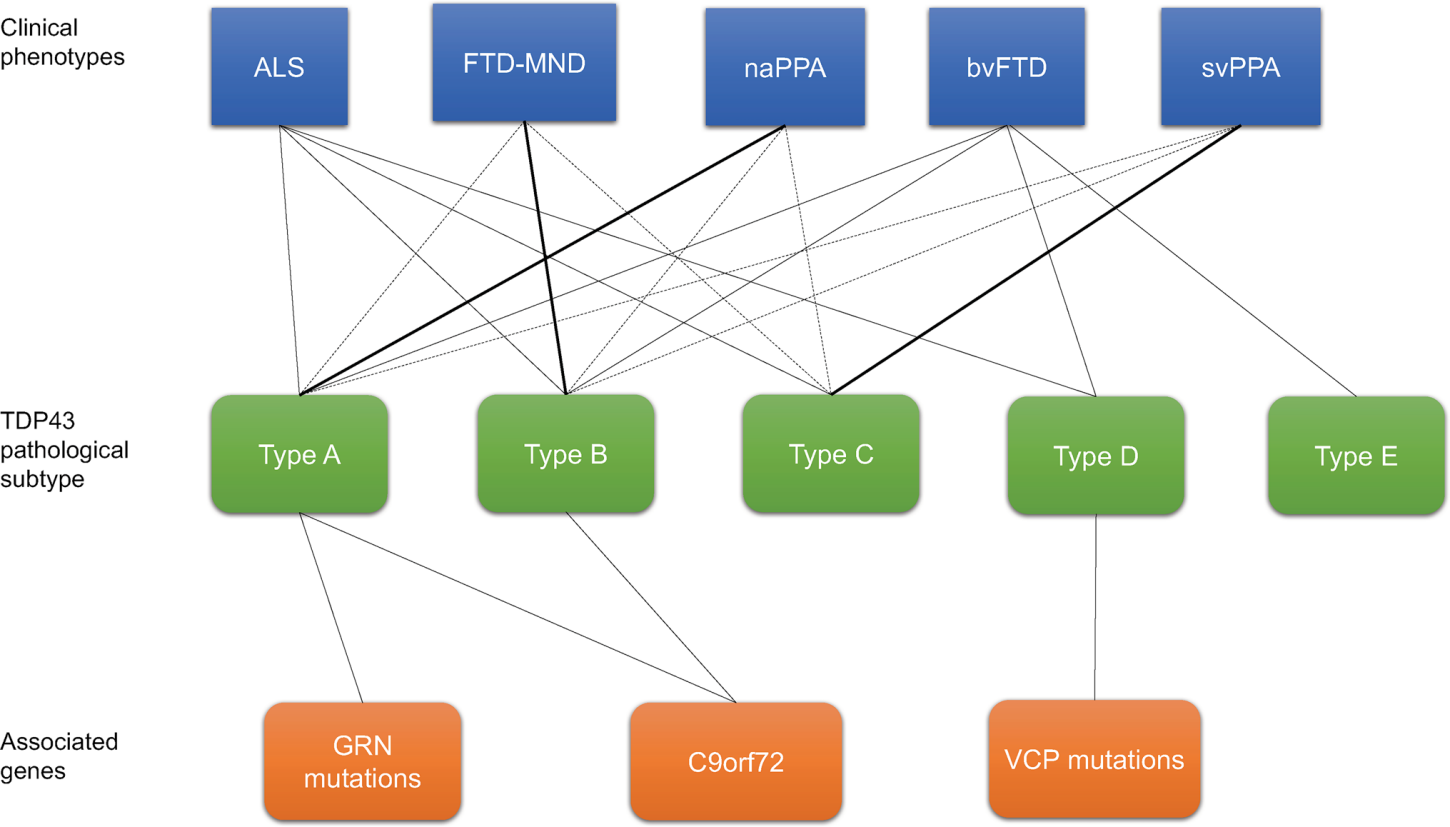
Clinical, pathological, and genetic spectra of TDP proteinopathy. *ALS* amyotrophic lateral sclerosis, *bvFTD* behavioral variant frontotemporal dementia, *MND* motor neuron disease, *naPPA* nonfluent/agrammatic PPA, *svPPA* semantic variant PPA, *GRN* granulin, *VCP* valosin-containing protein

Another study classified TDP-43 pathology in non-FTLD brains into two subtypes, TDP type-α and TDP type-β. The former shows typical TDP-43 immunoreactive inclusions, while the latter is characterized by the presence of TDP-43 immunoreactivity adjacent to/associated with NFTs in the same neuron [81]. The age at death of type-α was older than that of type-β (median 89 years vs. 87 years). Hippocampal sclerosis was present in 60% of type-α patients and 15% of type-β patients. A pattern of widespread TDP-43 deposition commonly extended into the temporal, frontal and brainstem regions in type-α, whereas TDP-43 deposition was predominantly located in limbic regions, including amygdala, entorhinal cortex and subiculum of the hippocampus in type-β [81]. The frequency of *TMEM106B* protective (GG) and risk (CC) haplotypes (SNP rs3173615 encoding p.T185S) is high in type-α compared to type-β in non-FTLD-TDP cases [81] (Fig. 6).

Recently, the new term limbic-predominant age-related TDP-43 encephalopathy (LATE) was proposed to describe the presence of TDP-positive lesions in AD, as well as in older adults [112]. LATE neuropathological change (LATE-NC) is defined by a stereotypical TDP-43 proteinopathy in older adults, with or without coexisting hippocampal sclerosis pathology, and is distinguished from FTLD-TDP. LATE-NC is also a common TDP-43 proteinopathy associated with an amnestic dementia syndrome similar to AD. LATE includes already characterized phenotypes, but involves no new TDP-43 pathologic subtype, no link between TDP pathology with new cognitive symptoms, and no characteristic biochemistry [79]. Although the term aims to encourage future research, as well as development of neuroimaging and molecular biomarkers, the concept may be confusing in relation to AD and should be considered further.

## Genetic predisposition leading to differential vulnerability

The pathological classification of TDP-43 proteinopathy is supported by clinical and genetic correlations. svPPA is associated with type C, and FTLD-MND or clinical signs of MND with type B, and naPPA with type A, although bvFTD is not as strongly correlated with any special pathological type [21, 78, 85, 100]. Most cases of ALS/FTLD are sporadic but ~ 5% is familial. Regarding the genetics, clinical clusters of FTD and ALS have been reported to be associated with several genes, including *granulin precursor* (*GRN*) [15, 42], *valosin*-*containing protein* (*VCP*) [153], *TAR DNA*-*binding protein* (*TARDBP*) [85, 91], *chromosome 9 open reading frame 72 gene* (*C9orf72*) [45, 100, 124], *sequestosome 1* (*SQSTM1*) [148], *ubiquilin 2* (*UBQLN2*) [46], *TANK*-*binding kinase 1*(*TBK1*) [56], *T cell*-*restricted intracellular antigen*-*1 gene* (*TIA1*) [103]*, cyclin F* (*CCNF*) [158]*, coiled*-*coil*-*helix*-*coiled*-*coil*-*helix domain*-*containing protein 10* (*CHCHD10*) [16] and optineurin (*OPTN*) [35]. Superoxide dismutase 1(*SOD1*) has also been linked with ALS [128]. *C9orf72* repeat expansion is the most frequent genetic factor in sporadic and familial cases [2, 33, 66], but 40% of familial cases have no established genetic cause, and unknown genes may be involved [2, 66]. Cases with a *C9orf72* expansion repeat mutation have additional p62-positive and TDP-43-negative NCIs, which are composed of dipeptide repeat proteins (translated from the *C9orf72* expansion repeats) [100]. Psychotic symptoms, such as delusions and hallucinations, are likely to present in *C9orf72* repeat expansion carriers [47, 138], although they are generally rare in FTD [97]. Also, family members of patients carrying *C9orf72* repeat expansion might have high incidences of psychiatric illness [47]. *GRN* is mostly associated with type A, and *C9orf72* with type B, or less commonly with type A [100, 101]. Associated genes are still unknown in types C and E [95]. Type D has been reported to be highly associated with inclusion body myopathy with early-onset Paget disease of bone and frontotemporal dementia (IBMPFD) caused by VCP mutations. Missense mutations in VCP affect various cellular processes, including ubiquitin-dependent protein quality control, cell division, and nuclear envelope formation [110]. As a result, the consolidation of aggregate-prone proteins into inclusion bodies via multivesicular body formation is induced and causes the pathogenic features [149].

Various related genes, apolipoprotein E (*APOE*), granulin (*GRN*), transmembrane protein 106B (*TMEM106B*), ATP-binding cassette sub-family member 9 (*ABCC9*) and potassium channel subfamily M regulatory beta subunit 2 (*KCNMB2*), have been reported to be risk alleles associated with pathological manifestations in FTLD-TDP and LATE [112].

## Progression pattern of TDP-43 pathology in human brains

Clinical phenotypes are associated with characteristic distribution patterns of the pathology in the course of disease progression. The progression pattern of TDP-43 pathology has been identified [26, 27]. In bvFTD, pTDP-43 pathology progresses in four distinct topographic stages [26]. Stage I involves the orbital gyri, gyrus rectus, and amygdala, and stage II progresses to the middle frontal and anterior cingulate gyrus as well as anteromedial temporal lobe areas, the superior and medial temporal gyri, striatum, red nucleus, thalamus, and precerebellar nuclei. Stage III shows involvement of the motor cortex, bulbar somatomotor neurons, and the spinal cord anterior horn, whereas finally, stage IV shows the highest burden of pathology in the visual cortex [26]. bvFTD is associated with all subtypes of TDP pathology. In the case of svPPA with type C, the progression pattern of TDP-43 pathology is not yet established.

**Fig. 7:**
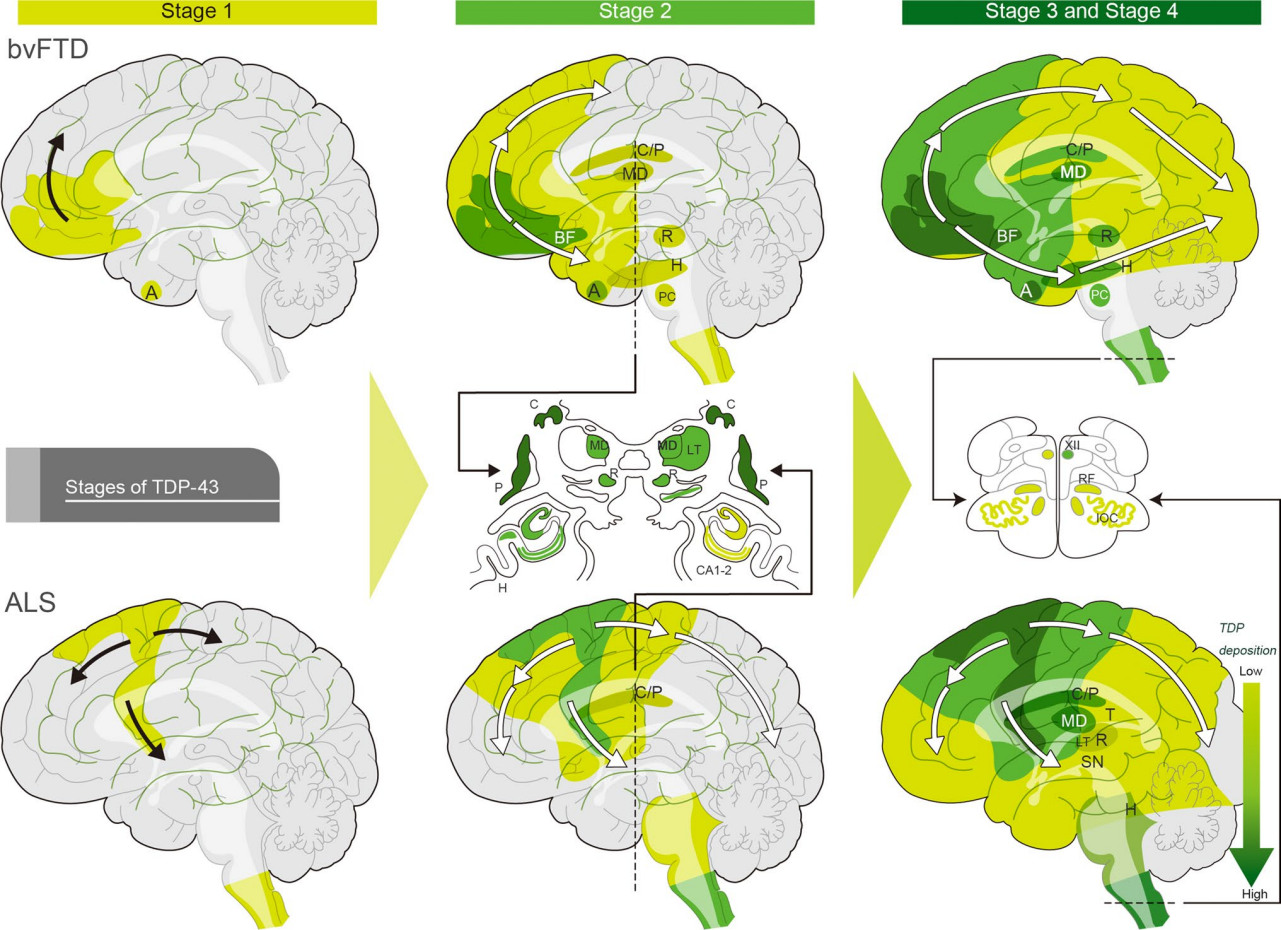
Progression of TDP pathology in bvFTD and ALS The upper figure shows TDP-43 pathology progression in behavioral variant-frontotemporal dementia (bvFTD) of Types A and B, possibly propagating along axonal pathways. Stage 1: pTDP-43 pathology begins with neurons and oligodendrocytes of the basal and anterior portions of the prefrontal neocortex and amygdala. Stage 2: the pathology appears caudal to the frontal lobe, including the middle frontal gyrus, insular cortex, and anterior cingulate gyrus, and also in anteromedial areas, including the hippocampus (H), the caudate nucleus and putamen (C/P), and the mediodorsal nucleus (MD) of the thalamus (T), red nucleus (R), precerebellar nuclei (PC), and dorsomedial medullary regions. Stage 3: TDP pathology is increased compared to the first two stages and has spread further into the cortical, brainstem, and spinal motor regions. Stage 4 cases with the highest burden of pathology, showing pTDP-43 deposits in the occipital neocortex (visual cortex, Brodmann 17, 18) Amyotrophic lateral sclerosis (ALS) with TDP-43 pathology is shown in the lower figures. Stage 1: pTDP-43 deposition begins (stage 1) in the agranular motor cortex, or lower motor neurons in the spinal cord and lower brainstem. Stage 2: increasing burden of TDP-43 pathology, with progression to the posterior frontal and anterior parietal regions, brainstem reticular formation (RF), PC and R. Stage 3: TDP pathology involves the anterior frontal and basal forebrain, the C/P, MD and lateral thalamus (LT), and substantia nigra (SN), Stage 4: TDP pathology has spread into the anterior temporal lobe, including the *H. cornu* ammonis subregions 1 and 2 of the hippocampus (CA1–2), hypoglossal nucleus (XII), and inferior olivary complex (IOC) Figures are adapted from Brettschneider et al. with permission from the copyright holder [26, 27]

The progression pattern of pTDP-43 in ALS patients has also been characterized [24, 27]. ALS with TDP deposition begins in the upper motor neurons in the cortex, lower motor neurons in the spinal cord and lower brainstem (stage I). Stage II progresses gradually to posterior frontal and anterior parietal regions, brainstem reticular formation, and red nucleus. Stage III involved the anterior frontal and basal forebrain, striatum, thalamus (mediodorsal/lateral thalamus), and substantia nigra. Finally, patients show involvement of the anterior temporal lobe including hippocampus (stage IV) [24, 27] (Fig. 7).

## Methods for assessing clinical phenotypic diversity

Several methods have been developed with the aim of identifying the differences between TDP-43 and other proteinopathies. Many researchers have investigated TDP-43 in cerebrospinal fluid (CSF) since it is considered to reflect the TDP pathology in human brain. However, the results are conflicting. One report found that ALS and FTLD patients had higher TDP-43 levels in CSF, compared to control patients [141], but another report indicated that TDP-43 CSF levels were higher in ALS, in comparison with FTLD [84]. In the latter paper, there was a difference in *C9orf72* mutation between ALS and FTLD patients, but no difference was detected between carriers and non-carriers. Furthermore, a non-significant trend of lower pTDP-43 levels in ventricular CSF was reported in FTLD-TDP compared to controls [92]. These results suggest that TDP-43 CSF level does not adequately reflect TDP-43 pathology, and may not be a useful biomarker [140]. Further, TDP-43 analysis in blood is not yet available for the diagnosis of TDP-43 pathology, due to the lack of sufficiently sensitive quantification methods [53, 54]. However, more advanced methods should be developed in the near future.

**Fig. 8:**
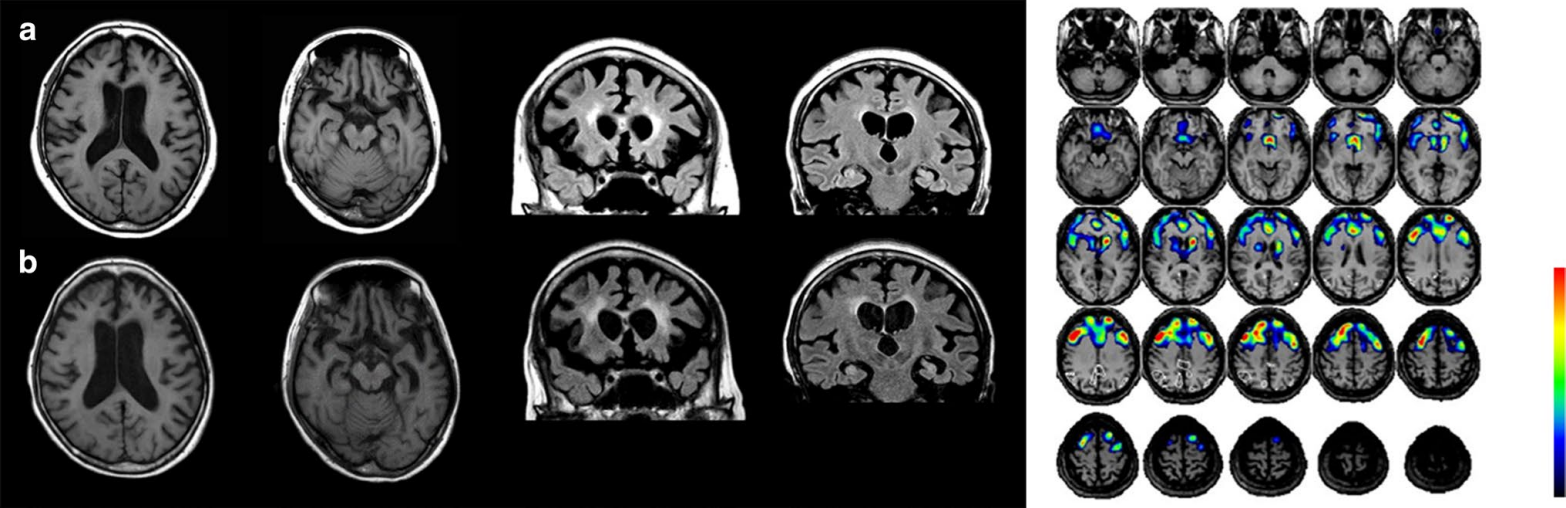
Imaging of an FTLD-TDP patient showing the clinical presentation of naPPA. Left photo shows T1-weighted MRI at onset (**a**) and 2 years after onset (**b**). Frontal and temporal atrophy on axial slices (two photos on the left) is prominent. Coronal slices (two photos on the right) indicate atrophy in the dorsolateral prefrontal cortex and medial and inferior temporal cortex with enlargement of the temporal horn. Single-photon-emission computed tomography using technetium-99 m ethyl cysteinate dimer (ECD-SPECT) revealed a reduction in the bifrontal regions (Right photo)

Several neuroimaging studies of FTLD-TDP and ALS have been reported. Volumetric structural MRI scans, cortical thickness analyses, diffusor tensor imaging, and to a lesser extent functional techniques, including functional MRI and FDG-PET have been utilized to aid diagnosis in clinical practice [20, 33]. Generally, patients with FTLD classically show frontal and temporal atrophy, involving the orbitofrontal/dorsolateral prefrontal cortices and the temporal poles, and hypometabolism in these regions is often asymmetrical. As mentioned above, there are characteristic patterns of atrophy in the three clinical phenotypes of FTLD. MRI or positron emission tomography (PET) findings have revealed frontal and/or anterior temporal atrophy or hypometabolism in bvFTD [123], left posterior frontoinsular atrophy, hypoperfusion or glucose hypometabolism in naPPA, and predominant anterior temporal lobe atrophy or hypoperfusion or hypometabolism in svPPA [64, 129]. In the diagnosis of ALS, imaging is used only for exclusion of other diseases [28] (Fig. 8).

Imaging studies indicate that neurodegenerative diseases are caused by degeneration within specific intrinsic functional connectivity networks vulnerable to their pathologies [132, 165]. fMRI studies in bvFTD show attenuated connectivity within an anterior network of dorsal anterior cingulate and frontoinsular cortices connecting to subcortical and limbic structures [131, 166] and network disruption, with reduced mean network extent and increased path length, compared with healthy controls [1]. These results enable us to recognize sites of early disruption as vulnerable regions, and also throw light on the connectivity of the areas involved in the diseases. Further molecular pathological approaches will help to establish which aberrant proteins progress along networked brain structures, and whether specific pathological subtypes can be linked to specific forms of neural network degeneration [52].

Since the discovery of *C9orf72* in 2011, the role of the repeat expansion in TDP-43 proteinopathy has been investigated by means of imaging techniques. Recent neuroimaging analyses demonstrate that patients with *C9orf72*-positive bvFTD show symmetric atrophy most prominently in the anterior insula, anterior cingulate, and frontotemporal cortex, in keeping with the sporadic bvFTD-associated pattern [23, 75, 104, 133, 155]. Some studies indicate that *C9orf72* carriers exhibit atrophy of the parietal lobe [75, 155] and occipital lobe [133, 155], which are not typically involved in bvFTD. A resting-state functional MRI study found that patients with bvFTD with or without *C9orf72* expansion show convergent large-scale network breakdowns despite having distinctive atrophy patterns [96]. In ALS, extensive cortical and subcortical frontotemporal involvement has been reported in association with the *C9orf72* genotype, compared to the relatively limited extramotor pathology in patients with *C9orf72*-negative ALS [19]. The changes in the orbitofrontal, fusiform, thalamic, superior temporal regions, and Broca area, and posterior cingulate in ALS are distinct from those seen in the *C9orf72* genotype [19]. As for PET, *C9orf72*-positive ALS had a more widespread central nervous system involvement than *C9orf72*-negative ALS, with or without FTD comorbidity [38].

## Strains and phenotypic diversity

### Selective neuronal and regional susceptibility

Understanding the underlying causes is crucial for building effective treatment strategies. As described, neurodegeneration in ALS selectively involves the upper and lower motor neurons within the pyramidal motor system [134]. Motor neurons are different from many other neuronal cells in both size and shape. In addition, characteristic anatomical features include a large cell body with a high level of mitochondrial activity and a long axonal process with a high content of neurofilament proteins [134]. Those anatomical features are associated with mRNA splicing, oxidative stress, proteosomal and mitochondrial dysfunction, and glutamatergic toxicity with damage to critical target proteins in the pathogenesis of ALS [65, 134]. Also, axonal cytoskeletal disorganization has been considered as a conspicuous feature of ALS. ALS linked with mutations in TDP-43 shows impairment of the axonal transport of RNA granules in animal models of TDP-43 proteinopathy [3].

TDP-43 accumulations are detected in unaffected cells in ALS, though some Betz cells, which mainly degenerate in ALS, are known to lack TDP-43 accumulation [25, 59]. This may imply that there is no direct correlation between the presence of the accumulations and cell death. The Betz cells in upper motor neurons of patients display a distinct set of intracellular defects, especially at the nuclear membrane, mitochondria and endoplasmic reticulum [59]. Another argument against the hypothesis is that only cells with TDP-43 accumulation cause degeneration. Previous data suggest that MND with ALS-linked mutants producing loss and gain of splicing function of selected RNA targets at an early disease stage does not require TDP-43 accumulation and loss of TDP-43 from the nuclei [10].

Many physiological and pathological studies indicate system degeneration in ALS and FTLD. ALS patients present focal clinical symptoms showing upper or lower motor neuron predominance and clear alterations in motor function in one body region at a specific time, typically within a timescale of weeks to months [142]. The neuroanatomical distribution of pTDP-43 pathology allowed classification of FTLD cases into several clinical subtypes, as described above. Thus, the disease does not progress randomly across contiguous anatomical pathways [142]. It is important to consider the pattern of progression of neurodegeneration at the macroscopic level, in addition to the selective neuronal vulnerability at the microscopic level.

The vulnerable circuits and cell types in FTD have recently been investigated. Especially in bvFTD, von Economo neurons and fork cells within the anterior cingulate and ventral anterior insular cortices are affected in early degeneration [88, 111]. The large layer 5 projection neurons in the regions undergo early dropout in bvFTD [88, 111]. In addition, the presence of TDP-43 inclusions was associated with striking nuclear and somatodendritic atrophy and the inclusion fraction in those cells correlated with symptom severity, such as loss of emotional empathy, in bFTD [111]. These insights regarding selective vulnerability and the mode of their progressive degeneration might lead to the identification of new targets for therapy and effective treatments for numerous neurodegenerative diseases.

### Current evidence regarding strains and progression

The different banding patterns of abnormal TDP-43 fragments in ALS and FTLD may represent different TDP-43 strains with different conformations. Biochemical analysis of different brain regions and spinal cord in individual patients with TDP-43 proteinopathy revealed three distinct C-terminal banding patterns [40, 68, 147]. This strongly suggests that the same form of abnormal TDP-43 molecule is consistently deposited in different brain regions in each patient. It is unlikely that the same conformational change would occur synchronously in different brain regions, and it seems more likely that abnormal protein produced in cells is transferred to different regions, transmitted from cell to cell and propagated in vivo [40, 68, 69, 72, 147]. In addition, novel cell-based techniques indicate that there are distinct forms of pathological TDP-43 with different biochemical features, which correlate with the FTLD subtypes; they manifest distinct neurotoxicities and show different seeding activities in culture [40, 68, 72].

In prion diseases such as CJD and bovine spongiform encephalopathy, the different patterns of protease-resistant bands can be used to identify the etiology of the diseases [40, 120]. For example, protease-resistant prion from new variant CJD exhibits a different banding pattern from that in sporadic CJD cases, and the band pattern was indistinguishable from that of mice infected with bovine spongiform encephalopathy prion. These observations are consistent with the idea that the different banding patterns of abnormal TDP-43 fragments in ALS and FTLD might represent different TDP-43 strains with different conformations.

A hypothesis that has received much attention recently is that neurodegeneration might spread from cell to cell by a mechanism that is seen in prion diseases [117]. According to this hypothesis, abnormal insoluble TDP-43 in brains of patients exhibits prion-like properties. Recently, pathological TDP-43 derived from FTLD-TDP brains was shown to induce formation of de novo TDP-43 pathology with subsequent spreading throughout the central nervous system in a region- and time-dependent manner in experimental animal models [122]. It was also reported that TDP-43 could be released from cells via secreted vesicles called exosomes. This could facilitate the propagation of prion-like TDP-43 aggregates from one cell to others. The types of TDP-43 aggregates (strains) initially generated in neuronal/glial cells spread to the neighboring cells and determine the TDP-43 pathologies. One study found that inhibition of exosome secretion exacerbated the disease phenotypes of transgenic mice expressing human TDP-43 mutant [74]. Morphological analyses of mouse primary cortical neurons, patients’ fibroblasts, and stem cell-derived neurons showed that aggregation of TDP-43 triggered the sequestration and mislocalization of nucleoporins and transport factors, and interfered with nuclear protein import and RNA export [37].

### Exogenous and environmental factors modulating selective regional vulnerability

Clinical studies suggest that traumatic brain injury (TBI) might be associated with an elevated risk of ALS, as well as other devastating neurodegenerative diseases [125, 126]. Chronic traumatic encephalopathy (CTE) is a neurodegenerative disease caused by repeated TBI associated with contact sports, and diseased brains contain abnormal accumulations of hyperphosphorylated tau [106]. Head trauma induces focal axonal injury, microhemorrhage and gliosis in close proximity to the affected area, then p-tau accumulates in neurons in the perivascular regions due to axonal injury, breach of the blood–brain barrier, and neuroinflammation, and finally p-tau spreads throughout the brain [108]. A previous paper describes patients with repetitive head trauma who showed widespread TDP-43 inclusions in the CNS, including motor neurons [107]. A study in *Drosophila* models of ALS indicated that repetitive trauma promotes the accumulation of TDP-43, with SGs in the brain, and also exacerbates neurodegenerative phenotypes as indicated by mortality and locomotor dysfunction [4]. Reversible induction of TDP-43 in cortical neurons after traumatic injury was also observed in a mouse stab wound model of TBI [156]. A single instance of TBI might not necessarily cause TDP progression, but repetitive head trauma may initiate or modify the onset or progression of ALS [146]. The precise etiology remains unclear and further research is needed to elucidate the impact of trauma on TDP-43 propagation.

In terms of selective vulnerability, exposures outside the CNS may influence motor neurons themselves. Especially in the spinal cord, motor neurons lack the protection of the blood–brain barrier at the axon termini. The neuromuscular junction seems vulnerable and the retrograde transport of aberrant factors may be able to occur at this site in patients [142].

### Experimental modeling of phenotypic diversity

The development of multiple experimental models of TDP-43 proteinopathy is essential to clarify the pathogenic mechanism underlying TDP-43 propagation. Protease-resistant banding patterns of pathological insoluble TDP-43 are useful for biochemical classification of the subtypes [147]. Cell-to-cell transmission of TDP-43 aggregates also occurs in cell culture, and exosomes are likely to be involved in pathological TDP-43 propagation [51, 117, 135]. SH-SY5Y cells treated with insoluble TDP-43 extracted from ALS or FTLD-TDP brains expressed aggregates of phosphorylated and ubiquitinated TDP-43 in a self-templating manner [117]. Immunohistochemical analyses established that the C-terminal fragments of each disease type acted as seeds in these cells, inducing seed-dependent aggregation of TDP-43 [117]. The seeding ability of insoluble TDP-43 was hardly affected by protease treatment, but was abrogated by formic acid. Another study indicated that anterograde and retrograde transport of TDP-43 oligomers occurs in cultured cells, providing support for the idea of cell-to-cell transmission of pathological TDP-43 [51]. Furthermore, residues 274–353 are essential for the conversion of TDP-43 to amyloid-like fibrils, and aggregation of TDP-43 by seeding with different peptides induces various types of TDP-43 pathologies [135]. These findings indicate that insoluble TDP-43 has prion-like properties that likely play a role in disease progression of patients with TDP-43 proteinopathy.

As in the cases of tau and α-synuclein, the prion-like propagation of TDP-43 protein in vivo has been investigated by injecting brain extracts from aged transgenic mice showing pathology, or synthetic protein fibrils, or extracts from brains of patients into mouse brains. Animal models of TDP-43 propagation are not easy to prepare, compared to models of tau and α-synuclein propagation. It is very difficult to prepare substantial amounts of natively folded recombinant proteins or fragments in *E. coli*, and so it is difficult to form synthetic amyloid-like TDP-43 fibrils [70]. In addition, unlike α-synuclein or tau, TDP-43 is localized in the nucleus and its expression is tightly self-regulated [14], which may stabilize the conformation of TDP-43 in vivo and prevent the formation of abnormal TDP-43 pathology in non-Tg mice. However, more recent studies succeeded in the formation of TDP-43 pathology with subsequent spreading throughout the central nervous system in a region- and time-dependent manner by injecting pathological TDP-43 of FTLD-TDP into transgenic CamKIIa-hTDP-43NLSm mice, which overexpress mutant TDP-43 lacking a nuclear localization signal [122]. The propagation of TDP-43 pathology in the injected mice resulted in widespread cortical, hippocampal, and subcortical pTDP-43 accumulation in both ipsilateral and contralateral hemispheres, consistent with cell-to-cell transmission of TDP-43 pathology following the neuroanatomical connectome from the injection site. Another report clarified phenotypic heterogeneity among mutant mice and identified distinct transcriptomic profiles corresponding to differing phenotypes. The authors observed changes linked with improved behavior in TDP-43 knock-in mouse with a human *TARDBP* gene, associated with downregulation of two known modifiers of neurodegeneration, Atxn2 and Arid4a, and upregulation of myelination and translation genes. Identifying the environmental factors that influence the delicate balance in the transcriptome of the brain might be a promising topic for future studies [154]. Other physiological models indicate that aggregation and nuclear depletion are dispensable for neurodegeneration [55, 154]. Further work is necessary to establish the relationship between the seeding activity or strain-like properties of pathological TDP-43 in patients and the neuropathological phenotypes of the mice.

## Concluding remarks

Increasing evidence indicates that ALS/FTLD-TDP disease progression is induced by cell-to-cell propagation of intracellular aggregated proteins. Extensive biochemical and biophysical studies needed to clarify the pathomechanisms leading to the presence of distinct pathogenic TDP-43 strains in patients’ brains. Using genome editing technology, it should be possible to generate large knock-in animal models or human iPSCs to overcome overexpression, off-target, and mosaic effects, which could offer promising approaches for therapy [39]. In addition, cryo-electron microscopy (cryo-EM) will be a promising method to clarify the strains of pathological TDP-43. As for tau, a recent report has revealed the structures of the disease-specific folds in the ordered cores of tau filaments, establishing the existence of molecular conformers [49].

Genome-wide association studies have uncovered over 100 loci that are linked with ALS/FTLD [77]. Some ALS/FTLD-TDP patients have a familial history of neurological and/or psychiatric diseases that might possibly be associated with unknown genetic mutations. [14] Identifying those mutations would be helpful to identify the factors modulating disease progression and to provide targets for development of therapeutic interventions. Gene therapy or antisense oligonucleotide (ASO) therapy to knock out a relevant gene or to reduce expression of the protein might also be effective to prevent protein aggregation and prion-like propagation.

Recent years have seen a rapid development of fluid biomarkers for FTLD. In C9orf72 mutation carriers, the concentration of poly GP, which is produced as an abnormal dipeptide repeat protein, could be a target biomarker, in addition to TDP-43 [164]. The development of an immunoassay method for its detection in CSF will be needed.

Identification of imaging biomarkers, such as amyloid-imaging probes, that predict future clinical syndrome will be critical for the identification of suitable candidates for clinical trials. In addition, clinicopathological studies of TDP-43 are expected to provide deeper insights into disease mechanisms, although we should pay attention to the limitations involved in investigating patients with end-stage disease. Such studies should provide further support for the idea that clinical heterogeneity in patients is associated with the heterogeneity of TDP-43 neuropathology resulting from different TDP-43 strains.

Small molecular compounds that can pass through the blood–brain barrier, enter the cells, and bind to intracellular aggregates of abnormal proteins would be promising candidates for pharmacotherapy of neurodegenerative diseases by promoting clearance of pathological TDP-43 proteins, e.g., by activating degradation or protein quality-control systems. Antibody therapy may also be feasible.

In conclusion, combinations of behavioral, functional, and physiological approaches to study the changes in the neuroanatomical system will be crucial for the development of future therapy. In addition, the clinically stereotyped nature of the disease progression, and neuronal brain imaging studies, suggests that a system level vulnerability is a significant aspect of the biological underpinning of TDP-43 proteinopathy.
